# Preliminary effectiveness of the youth empowerment digital intervention (YEDI): A pilot clustered community study of a group cognitive behavioral therapy intervention with digital reinforcement among youth in Kenya’s informal settlements

**DOI:** 10.1017/gmh.2026.10278

**Published:** 2026-07-21

**Authors:** William Byansi, Catherine Mawia Musyoka, Christopher E Baidoo, Michael Galvin, Wilberforce Kurevakwesu, Moses Okumu, Sung Seek Moon, Maurice A Kalande, Muthoni Mathai

**Affiliations:** 1 School of Social Work, Boston College, USA; 2 Department of Psychiatry, University of Nairobi College of Health Sciences, Kenya; 3 Department of Behavioral Health Science and Practice, University of South Florida, USA; 4 School of Social Work, University of Illinois Urbana-Champaign, Urbana-Champaign, USA; 5 School of Social Sciences, Uganda Christian University, Mukono, Uganda; 6 Diana R. Garland School of Social Work, Baylor University, USA; 7 Fountain of Hope Treatment Center, Kenya

**Keywords:** alcohol use, cannabis use, mental health, urban informal settlements, cognitive behavioral therapy

## Abstract

Substance use and mental health symptoms frequently co-occur among youth in Nairobi’s informal settlements, yet scalable interventions remain limited. This pilot study evaluated the Youth Empowerment Digital Intervention (YEDI), a brief group cognitive-behavioral therapy model supported by mobile reinforcement. A clustered-community randomized pilot study enrolled 94 adolescents and young adults aged 15–24 years (treatment = 49; control = 45). The intervention group received seven weekly 1-hour manualized group cognitive-behavioral therapy sessions supplemented by mobile skill reinforcement, while control participants received standard health materials. Alcohol, cannabis, and tobacco use risk, along with depression, anxiety, and stress symptoms, were assessed at baseline, 3 months, and 6 months using linear mixed-effects models. YEDI demonstrated promising short-term reductions in alcohol risk, cannabis risk, and depressive symptoms at 3 months, although these differences were not sustained at 6 months. Tobacco risk did not differ between groups, and stress showed counterintuitive relative effects due to reductions in the control group. Delivering YEDI in informal settlements is feasible and provides a proof of concept for brief, early mental health support. Preliminary findings indicate that brief, skill-based models alone cannot offset severe structural stressors. Multi-level interventions integrating behavioral strategies with ongoing community support are required. .

## Graphical abstract

**Figure:**
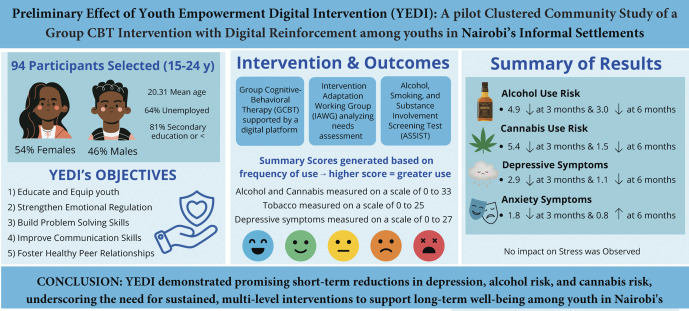

## Impact statement

Adolescents and young adults living in informal urban settlements face a convergence of structural adversity, including poverty, violence exposure and limited access to mental health and substance use services. Despite the high burden of co-occurring psychological distress and substance use in these contexts, there remains a critical shortage of scalable, evidence-based interventions that are feasible to deliver in resource-constrained community settings. This study provides preliminary evidence on the Youth Empowerment Digital Intervention (YEDI), a brief, community-delivered group cognitive-behavioral therapy program complemented with a digital app, designed specifically for youth in Nairobi’s informal settlements. The findings show that YEDI reduced depressive symptoms and alcohol and cannabis use risk at 3 months, proof-of-concept for task-shared and digitally reinforced delivery in informal settlement contexts. These results suggest that brief individual-level skills interventions may be helpful but insufficient on their own in settings shaped by poverty, violence exposure, unstable employment and schooling and limited access to health and mental services. Future intervention models should integrate behavioral skills with structural supports, community referral systems, youth livelihoods or education supports and sustained follow-up. Because the study did not isolate the digital component, findings should not be interpreted as evidence that digital delivery independently improved outcomes. Overall, this pilot establishes proof of concept for YEDI’s delivery model and provides the effect estimates needed to design and power a fully resourced, multi-cluster trial.

## Introduction

Alcohol, cannabis and tobacco use, along with depression, anxiety and stress, constitute a significant disease burden among adolescents and young adults globally, with mental health conditions alone accounting for up to 20% of the health burden among youth (Kessler et al., 2005; Kessler et al., 2007). This global trend is acutely amplified in vulnerable populations, such as those in Africa, where the burden of depression and anxiety among adolescents aged 10–19 years is particularly high (Jörns-Presentati et al., 2021; Piao et al., 2022). A recent systematic review and meta-analysis found that approximately one in four adolescents tried alcohol at some point in their lives and more than one-third had used alcohol within the past year (Belete et al., 2024). According to the National Authority for the Campaign Against Alcohol and Drug Abuse (NACADA, 2022)), Kenya faces similar concerns, with national estimates indicating that 8.9% of youth aged 15–24 years meet criteria for alcohol or substance use disorders, and 5.2% report current alcohol consumption. Young people in informal urban settlements face even greater risks due to poverty, overcrowding, limited services and high exposure to violence (Hatcher et al., 2019; Friedberg et al., 2023; Kibichii and Mwaeke, 2024; Kurevakwesu et al., 2024). These structural disadvantages create a fertile ground for exacerbated mental health and substance use issues. Among adolescents in Nairobi’s informal settlements, 9.2% reported moderate to severe depression, and 17.6% reported anxiety (Friedberg et al., 2023). In a recent study, youth with three or more adverse childhood experiences (ACEs) reported significantly higher depression and anxiety symptoms, underscoring the severe mental health burden among adolescents living in informal urban settlements (Byansi et al., 2025). Understanding why this burden is so concentrated in informal settlements requires examining the multiple risk pathways through which poverty, violence and social disadvantage shape substance use and mental health among young people.

Youth alcohol use and mental health symptoms emerge from multiple intersecting social, environmental and individual factors (Kurevakwesu et al., 2024; Perry et al., 2024), which are often exacerbated in settings like Nairobi’s informal settlements. At the individual level, impulsivity, sensation seeking and association with substance-using peers increase risk (Trucco, 2020). Family instability, exposure to violence and limited parental monitoring increase vulnerability to substance use and mental health problems. Community-level risks, such as neighborhood violence, drug availability, unemployment and weak institutional support, are primarily concentrated in informal urban settlements in Kenya (Odongo and Donghui, 2021; Kibichii and Mwaeke, 2024). Structural adversities, including poverty, school dropout and unstable housing, not only compound psychological distress but also significantly increase the likelihood of alcohol, cannabis and tobacco use among youth, often as maladaptive coping mechanisms (Friedberg et al., 2023). While protective factors such as strong family cohesion, peer support and access to safe recreational spaces can mitigate these risks, these crucial resources are frequently weak or inconsistent in informal urban settlements, highlighting a critical vulnerability that existing interventions often fail to address. Research on ACEs shows that early adversity sharply elevates risks for depression and anxiety, suggesting that structural hardship and early trauma intersect to shape harmful trajectories for young people (Kessler et al., 2010; Byansi et al., 2023; Byansi et al., 2025). Yet despite this well-documented accumulation of risk, the mental health and substance use service landscape in Africa remains inadequate to respond to the needs of young people in informal settlements.

Despite the high burden, few evidence-based interventions exist for youth mental health and substance use in SSA and access to services is extremely limited (Jaguga and Kwobah, 2020; Mabrouk et al., 2022; Kurevakwesu et al., 2024). Most mental health care is concentrated in urban referral hospitals and is hindered by high costs, long travel distances, limited workforce capacity and stigma surrounding mental illness and substance use (Jaguga and Kwobah, 2020; Too et al., 2021; Mabrouk et al., 2022). Youth in urban informal settlements rarely receive timely screening or treatment for alcohol and substance use or mental health problems (Perry et al., 2024). Existing programs are often not youth-centered, culturally adapted or designed for scalability. Research on co-occurring substance use and mental health symptoms in these settings remains limited, often relying on small, cross-sectional samples, single-site designs and inconsistent measurement tools (Jaguga and Kwobah, 2020). Moreover, existing studies often focus on prevalence rather than intervention effects or longitudinal changes (Too et al., 2021), further limiting the evidence base for effective strategies. These gaps highlight the urgent need for rigorous, context-appropriate intervention research targeting both substance use and mental health, which this study aims to address.

Psychosocial and brief alcohol interventions have shown promise in SSA, but the evidence base remains uneven. Reviews of brief interventions and psychosocial approaches suggest potential improvements in alcohol abstinence and reductions in harmful use, particularly in health-care or task-shared settings, although effects on alcohol use risk, drinking frequency and longer-term outcomes are inconsistent (Klimas et al., 2018; Sileo et al., 2020; Sileo et al., 2021). Scoping reviews of adolescent mental health interventions in SSA also show increasing use of lay-provider and community-based delivery models, but many programs have not been tested with rigorous longitudinal designs or adapted specifically for youth living in informal settlements (Too et al., 2021; Mabrouk et al., 2022). In Kenya, youth-focused screening and brief intervention approaches have been adapted for peer delivery, indicating the promise of task-shared substance use responses, but more evidence is needed on sustained outcomes and integration with mental health care (Jaguga et al., 2023). YEDI builds on this emerging evidence by combining group CBT skills, task-shared delivery and mobile reinforcement for youth experiencing mild-to-moderate substance use risk in informal settlements.

This study evaluates the preliminary longitudinal impact of a youth-focused group cognitive-behavioral therapy (GCBT) intervention designed to reduce alcohol and substance use and improve behavioral health outcomes in urban informal settlements. GCBT is a structured, skills-focused intervention informed by Social Cognitive Theory, which emphasizes how thoughts, emotions and behaviors are shaped through learning and reciprocal interactions with the environment (Bandura, 1986). From this perspective, substance use and related mental health symptoms can function as learned coping responses to stress and adversity, especially in high-risk contexts. GCBT targets these mechanisms by helping youth identify maladaptive cognitions, build self-efficacy and develop healthier behavioral strategies to manage challenges and reduce reliance on alcohol, cannabis and tobacco use. YEDI was conceptualized as an early intervention and secondary prevention program targeting adolescents and young adults with mild-to-moderate substance use risk and co-occurring emotional distress, rather than a clinical treatment intervention for severe substance dependence or acute psychiatric disorders. Depression, anxiety and stress were included as secondary outcomes because the intervention’s CBT components, including psychoeducation, relaxation, cognitive restructuring and problem-solving, target transdiagnostic coping processes relevant to both substance use and common mental health symptoms. Therefore, we hypothesize that adolescents and young adults receiving the intervention will show (a) greater reductions in alcohol, tobacco and cannabis use risk; and (b) greater reductions in depressive, anxiety and stress symptoms over time compared to their peers in the control condition. This study addresses critical limitations in SSA’s youth mental health and substance use literature by employing a longitudinal design, incorporating validated measures and examining both substance use and mental health symptoms simultaneously. The findings have the potential to inform scalable, evidence-based strategies to improve youth well-being in informal urban settlements across sub-Saharan Africa, where services remain severely constrained.

## Materials and methods

### Sample

This study draws on longitudinal data evaluating the impact of a youth-focused GCBT intervention (hereafter referred to as YEDI) designed to reduce alcohol and substance use and improve behavioral health outcomes. The study was implemented in two large informal settlements in Nairobi, Kenya. The trial employed a clustered community design in which two settlements were randomly assigned to either the control or intervention arm of the study. Mukuru Kwa Reuben and Kibra were selected because both are large informal settlements in Nairobi with established youth-serving partners, overcrowding, high levels of economic instability, limited health access to formal behavioral health services and high levels of social stressors, which provide an important setting for examining substance use and mental health outcomes among adolescents and young adults (Odongo and Donghui, 2021; Kibichii and Mwaeke, 2024). Settlement-level assignment was used to reduce contamination because participants lived in dense social networks, and intervention materials could be shared among peers. This study was designed as a pilot clustered community trial intended to support intervention refinement and estimation of preliminary effects rather than definitive cluster-level causal inference. Consequently, findings should be interpreted as exploratory and hypothesis-generating, and future studies should incorporate more clusters to enable more rigorous estimation of intervention effects and between-community variability.

Data were collected from a community sample between September and December 2024 using a snowball sampling strategy led by three trained community health promoters (CHPs). This method was chosen for its effectiveness in reaching hidden or hard-to-access populations within informal settlements. The CHPs selected three initial participants, two males and one female, who subsequently referred peers exhibiting similar behaviors for recruitment. These youth leaders, who were familiar with local patterns of alcohol and substance use, first identified peers known to use substances and then referred additional youth to participate. A total of 94 adolescents and young adults aged 15–24 years were recruited. Eligible participants were adolescents and young adults aged 15–24 years who had lived in the study community for at least three months and provided informed consent to participate in the behavioral health screening survey. For those ages 15–17, written consent from a parent or guardian and youth assent were required. Participants also needed to score below 15 on the Alcohol, Smoking and Substance Involvement Screening Test (ASSIST), indicating mild to moderate substance use, and have access to a mobile phone to enable follow-up assessments. There was no minimum ASSIST-Y score for inclusion. The ASSIST-Y was used to characterize risk and to exclude youth with severe risk scores who require a higher level of care. Depression was not used as a recruitment criterion. It was included as a secondary outcome because YEDI targeted shared cognitive and behavioral mechanisms, such as coping, stress regulation and maladaptive thoughts, that are relevant to both substance use and depressive symptoms. Both in-school and out-of-school youth were eligible to reflect the diverse experiences of adolescents and young adults in informal settlements.

Youth were excluded if they fell outside the 15–24 age range, had lived in the community for fewer than three months, scored 15 or higher on the ASSIST indicating severe substance use or presented with mental health symptoms requiring inpatient management. Individuals who were only temporarily residing in the area and were unlikely to remain for the duration of the study were also excluded. Those identified with severe substance use concerns were referred to government health facilities for appropriate care. Baseline assessments were completed before the initiation of any intervention activities, and participants were followed at 3 months and 6 months to examine changes in substance use and mental health symptoms over time.

Although snowball sampling increased access to hard-to-reach youth populations, we recognize that this approach may introduce selection bias, network homogeneity and overrepresentation of socially connected participants. To reduce these risks, recruitment was initiated through multiple community entry points and supported by youth-serving organizations and CHPs familiar with different areas of the settlements. Nevertheless, the non-probability sampling approach and two-community clustered design limit representativeness and the ability to disentangle intervention effects from site-level influences.

### Procedures

Five research assistants (RAs) with undergraduate training in psychology, public health or related fields were competitively hired and completed certification in Good Clinical Practice through the NIDA Clinical Trials Network. They also received intensive in-person training on confidentiality, interviewer safety, trauma-sensitive data collection and management of sensitive disclosures related to substance use and mental health.

Recruitment was facilitated through partnerships with two youth-serving community-based organizations. These two organizations served as the local implementation partners, providing community entry points, safe meeting spaces and logistical support. CHPs and youth mobilizers affiliated with these organizations distributed study information and identified potentially eligible youth through peer networks. RAs then independently conducted eligibility screening, consent and assent procedures and surveys. Thus, organizations supported recruitment infrastructure, CHPs supported peer referral and RAs retained responsibility for standardized research procedures. CHPs were instructed to identify adolescents and young adults aged 15–24 years who lived in the study communities and were known within the community to have recent mild-to-moderate alcohol or substance use behaviors. Depression, anxiety and stress symptoms were not used as recruitment criteria and were not described to CHPs as conditions for referral. CHPs were also not asked to determine severity. Instead, severity was assessed by trained RAs using the ASSIST-Y, and youth with scores indicating severe substance use risk were excluded and referred for care. Written informed consent was obtained from participants aged 18 and older, while assent and separate parental or caregiver consent were obtained for minors. All surveys were administered by trained RAs in English or Swahili, according to participant preference, and conducted in private settings to ensure confidentiality.

Participants received 500 Kenyan Shillings (5 USD) as compensation, in line with local research norms, to offset time and transportation costs. Privacy protections included secure handling of all identifiable data, strict adherence to confidentiality protocols and clear communication that participation was voluntary and could be discontinued at any time. Parents or guardians were not permitted to observe adolescent interviews. However, participants were informed that in the event of a life-threatening situation or reported child abuse, relevant authorities would be appropriately informed.

### Ethical review and oversight

The study protocol received ethical approval from the Kenyatta National Hospital and University of Nairobi Ethics Review Committee (KNH-UoN ERC No. P423/05/2024). We also received approval from the National Commission for Science, Technology and Innovation (NACOSTI/P/24/39331) in Kenya. All procedures adhered to established standards for research with human subjects. Participants who screened positive for severe psychological distress, suicidality or high-risk substance use were assessed by trained RAs under investigator supervision and referred to government health facilities per the approved crisis response protocol (Musyoka et al., 2025).

### Study conditions

**Control arm.** Adolescents and young adults in the control arm were not initially provided with the YEDI intervention. Control participants received the National Authority for Campaign Against Drug Abuse (NACADA) alcohol and substance use prevention materials, mental health literacy information and community self-help resources during the study period. These materials were also provided to the intervention arm to standardize basic information across conditions. The YEDI group sessions and mobile platform were available only to intervention participants during the 6-month follow-up period. Control participants were offered access to YEDI after the final assessment, which did not influence outcomes measured during the trial.

**Intervention arm** (YEDI). YEDI is a manualized Cognitive Behavioral Group Therapy program designed to address alcohol and substance use disorders, anxiety, stress and depressive symptoms among vulnerable adolescents and young adults. YEDI draws on core CBT mechanisms, such as cognitive restructuring, emotional regulation and problem-solving, to strengthen youth coping skills and enhance mental health (Klimas et al., 2018; Sileo et al., 2020; Sileo et al., 2021). Improved mental well-being is expected to contribute to healthier behaviors, including reduced substance use, improved interpersonal functioning, enhanced academic engagement and overall better quality of life.

Grounded in evidence-based principles (Sileo et al., 2020; Sileo et al., 2021), YEDI seeks to (1) educate youth about the effects of psychoactive substance use and common mental health disorders, (2) strengthen emotional regulation for healthier coping, (3) build problem-solving skills for responding adaptively to high-risk situations and (4) improve interpersonal and communication skills to support healthy relationships. The intervention prioritizes skill-building, peer support and experiential learning, similar to other successful CBT-based group interventions for youth.

### Structure and delivery of the YEDI intervention

YEDI was designed and developed by the Principal Investigator in collaboration with an Intervention Adaptation Working Group (IAWG) composed of researchers, mental health professionals, youth-serving providers and community stakeholders familiar with adolescent behavioral health challenges in informal settlements. The intervention development process was guided by the ADAPT-ITT framework (Wingood and DiClemente, 2008), a systematic model for adapting evidence-based interventions to new cultural and implementation contexts. Specifically, the team used the ADAPT-ITT phases of Needs Assessment, Decision, Administration, Production, Topical Experts, Integration, Training and Testing to guide adaptation of the intervention content and implementation approach.

YEDI was adapted from the Psychoeducation, Relaxation, Problem Solving, Activation, Cognitive Coping Therapy (PRO-ACT) that was led by one of the team members (see trial registration NCT06247527). The PRO-ACT was initially developed for adolescents and youth living with HIV in Kenya and was modified to address alcohol and other drug use and common mental health symptoms among adolescents and young adults living in Nairobi’s informal settlements. The IAWG developed a brief, manualized, skills-focused group intervention that incorporated psychoeducation about substance use and mental health, relaxation and stress management, problem-solving, cognitive restructuring, relapse prevention, communication skills and life skills training. The intervention content, training structure and implementation procedures were iteratively reviewed through role-plays, stakeholder consultations, and community feedback sessions. The adaptation process specifically focused on tailoring intervention scenarios, language and delivery approaches to the realities of youth living in informal settlements, including exposure to violence, unemployment, stigma, unstable housing and substance availability. For example, the team incorporated locally relevant examples related to peer pressure, cravings, cognitive coping and non-stigmatizing approaches to substance use screening and engagement.

YEDI consists of seven consecutive weekly sessions, each lasting approximately one hour. Sessions included psychoeducation, guided discussions, relaxation training, practice of cognitive and behavioral skills and group-based activities to encourage peer engagement and mutual support. The program was delivered in community spaces identified by partnering youth organizations, providing a familiar and accessible environment for participants. Participants were grouped by age and gender to ensure developmental appropriateness and psychological safety during group discussions. Four intervention groups were formed, each consisting of 8–10 participants. Two trained facilitators facilitated each group. We also developed a mobile platform (https://yedi.co.ke) for the YEDI intervention to reinforce skills learned during in-person sessions through mobile-based reminders, psychoeducational materials and behavioral health content accessible outside group sessions. The mobile platform was developed by a Kenyan designer and informed by the IAWG group. The team developed case scripts for the designer, which the IAWG reviewed. The digital platform featured brief animations to keep youth focused and engaged with the online content. All characters in the animation used pseudonyms suggested by the youth. Stakeholders, including youth participants, community members and researchers, reviewed the intervention materials and implementation strategy to identify barriers to engagement and to provide recommendations on facilitator selection, training logistics and acceptable approaches to intervention delivery in community settings.

**Figure 1.**
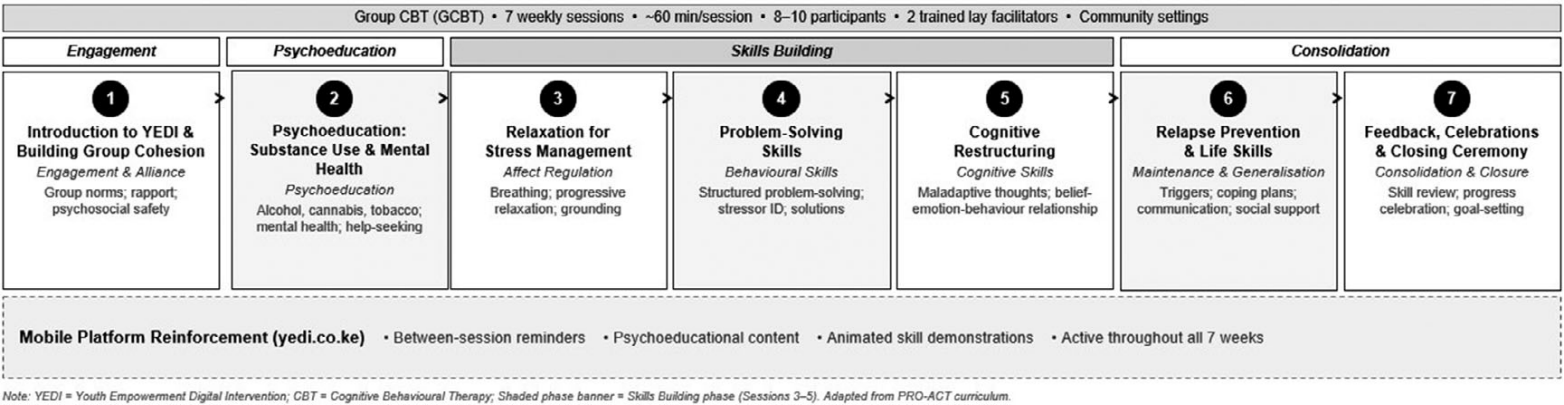
YEDI intervention session structure (7 weekly group CBT sessions). *Note:* CBT: cognitive behavioral therapy; shaded phase banner: skills building phase (Sessions 3–5); YEDI: Youth Empowerment Digital Intervention. Adapted from PRO-ACT curriculum. Figure 1. long description.

The content of the seven YEDI sessions is shown in Figure 1.

#### Recruitment, training and supervision of facilitators

Facilitators for the YEDI intervention were recruited from youth-serving organizations and community networks, prioritizing individuals with prior experience working with adolescents and familiarity with mental health and substance use issues in informal settlements. Twelve youth providers were selected and received standardized training delivered by two experienced trainers. Training content and expectations were refined through stakeholder engagement to ensure contextual relevance. Using the WHO EQUIP platform, facilitator competencies were assessed with the adapted ENACT-15 tool (Kohrt et al., 2015; Spedding et al., 2022), a validated measure designed to assess competencies in common therapeutic factors such as active listening, rapport building, confidentiality, empathy, risk assessment, collaborative goal-setting, psychoeducation and effective communication in low-resource settings. Each competency is paired with a structured role-play, rated on a four-level scale ranging from harmful behaviors to advanced skill demonstration.

Facilitators were youth peers aged 18–25 years who lived in the study communities and were identified with support from CHPs. They had prior experience participating in or supporting youth-serving programs in informal settlements and had completed at least secondary-level education (able to read and write in English). Facilitators were selected if they were of good standing in the community, possessed strong communication skills and were willing and interested in supporting community mental health initiatives. Before delivering the intervention, facilitators completed a 10-day training program covering group facilitation skills, communication techniques, confidentiality, handling sensitive topics, ethical conduct and the standard delivery of YEDI modules by the principal investigator (PI) and her mentors.

Training followed an Apprenticeship Model emphasizing active practice, iterative feedback and peer learning. Facilitators completed ENACT-based competency assessments (Kohrt et al., 2015; Spedding et al., 2022) at three time points: pre-training, post-training and post-supervision, using role-plays with standardized patient actors and vignettes developed and validated during the pilot phase. After training, facilitators delivered the YEDI sessions to small groups of youth (8–10 participants) presenting with mild to moderate psychological distress, under close supervision by a trained psychologist during the implementation phase. This supervision model supported fidelity in screening, session delivery and handling of sensitive mental health and substance use concerns.

## Measures

### Primary outcomes

#### Alcohol, cannabis and tobacco use

Alcohol, cannabis and tobacco use were measured using the World Health Organization Alcohol, Smoking and Substance Involvement Screening Test for Youth (WHO-ASSIST-Working-Group, 2002). The ASSIST-Y assesses lifetime and recent (past three months) substance use, cravings, use in risky situations and associated problems. Because the original ASSIST was designed for adults, the youth version omits items inappropriate for minors; however, the same tool has been validated for adolescents and young adults across multiple low-resource settings, including Kenya (Humeniuk et al., 2012; Jaguga et al., 2023). For each substance, summary scores were generated based on frequency of use, with higher scores indicating greater substance involvement. Alcohol and cannabis scores ranged from 0 to 33, and tobacco scores ranged from 0 to 25. These scores were analyzed continuously to capture variability across the full distribution. To standardize, the ASSIST-Y measure was administered to all participants regardless of age.

### Secondary outcomes

#### Depressive symptoms

Depression was assessed using the Patient Health Questionnaire-9 (PHQ-9) (Kroenke et al., 2001), a validated 9-item measure widely used in adolescent populations to assess depressive symptoms over the previous two weeks. Items are scored from 0 (“not at all”) to 3 (“nearly every day”), yielding a total score from 0 to 27, with higher scores indicating more severe depressive symptoms. The PHQ-9 has been validated for use in Kenya in both English and Swahili and demonstrates strong psychometric reliability (Osborn et al., 2022; Odero et al., 2023).

#### Anxiety symptoms

Anxiety symptoms were measured with the Generalized Anxiety Disorder-7 scale (GAD-7) (Spitzer et al., 2006). This 7-item measure asks respondents to rate the frequency of anxiety symptoms in the prior two weeks on a 0–3 scale. Total scores range from 0 to 21, with higher scores indicating elevated symptoms of anxiety. The GAD-7 has been validated in Kenya and has demonstrated good internal consistency among adolescents and young adults (Osborn et al., 2022; Odero et al., 2023).

#### Stress symptoms

Stress was assessed using the Perceived Stress Scale (Cohen et al., 1983), a widely used measure of perceived stress, unpredictability and lack of control in daily life. Participants rated 10 items on a 0–4 scale (“never” to “very often”), yielding a total score ranging from 0 to 40, with higher scores indicating greater stress. The PSS has been validated in diverse populations, including Kenyan samples (Manzar et al., 2019; Afulani et al., 2021).

### Control variables

Baseline control variables were assessed using a brief demographic questionnaire. Age was recorded in years; sex was self-reported (male/female). Education was categorized as secondary or less versus higher than secondary. Participants who completed secondary school but had no post-secondary training were classified as secondary or less. The higher-than-secondary group included any post-secondary education, including college, vocational or tertiary education. Living situation was self-reported and classified as living with a parent/guardian, living alone or living with a spouse. Employment status was self-reported and coded as working versus not employed.

### Power analysis

The power analysis was conducted a priori to guide sample planning. The primary purpose of this study was to test the pilot’s effectiveness; it was not powered to detect a predicted effect size for the continuous substance use outcomes. With two study sites and 48 participants per site, the sample provided sufficient precision to estimate means and variability in continuous ASSIST-Y alcohol, cannabis and tobacco risk scores. Using standard formulas for continuous outcomes and assuming an intraclass correlation (ICC) of 0.05–0.10, this sample size allowed us to estimate site-level mean substance involvement scores with reasonably narrow confidence intervals. Margins of error for mean estimates were expected to fall within the small-to-moderate range, supporting stable estimation of descriptive statistics and patterns of change over time. Because this pilot was not powered for hypothesis testing, the analytic focus was on estimating confidence intervals and patterns of change rather than detecting statistically significant differences. Overall, the sample size was adequate to characterize the distribution of continuous ASSIST-Y scores, inform future sample size calculations and examine preliminary longitudinal trends in substance use and mental health outcomes.

### Statistical analysis

We conducted all analyses in accordance with contemporary recommendations for handling longitudinal data with incomplete observations. Because missingness and attrition occurred across follow-up waves – particularly within the treatment arm – we addressed item- and wave-level missingness using multiple imputation with chained equations under a missing at random (MAR) assumption (Sterne et al., 2009). The MAR assumption was considered reasonable given that missingness was associated with observed characteristics such as treatment assignment and baseline outcomes. Consistent with best practice, the imputation model included all variables used in the analytic models, treatment condition, waves, all primary and secondary outcomes and demographic covariates, along with individual items contributing to each scale, thereby preserving scale-level distributions and maximizing efficiency (Van Buuren, 2018). We generated 30 imputed datasets, aligning with recommendations that 20–40 imputations help stabilize parameter estimates and reduce Monte Carlo error (Graham et al., 2007; Von Hippel, 2020).

Baseline demographic characteristics (age, sex, education level, living situation and employment status) were complete at Wave 1 for all participants and remained stable for participants observed at subsequent waves. Therefore, demographic values for Waves 2 and 3 were carried forward from baseline, given the absence of expected temporal variation in these characteristics over the study period.

We performed descriptive statistics appropriate to each variable’s level of measurement and examined baseline equivalence between the treatment and control groups using linear regression for continuous variables and logistic regression for binary variables.

Given the pilot nature of the study, analyses emphasized direction, magnitude and confidence intervals of group-by-wave estimates rather than hypothesis testing alone. To assess change in alcohol, cannabis and tobacco use, as well as depressive, anxiety and stress symptoms, we estimated linear mixed effects models (LMMs). Each model included random intercepts at the participant level to account for repeated measures nested within individuals, and fixed effects for wave, treatment group and the wave-by-treatment interaction. All demographic covariates were included as fixed effects. LMMs were selected because they flexibly accommodate unbalanced data, retain participants with partially observed outcome histories and adjust standard errors for within-person correlation. Parameter estimates and standard errors were pooled across all imputed datasets using Rubin’s rules (Rubin, 1987). We report unstandardized coefficients because outcomes were measured using interpretable, widely used scales, including ASSIST-Y, PHQ-9, GAD-7 and PSS, thereby allowing readers to directly interpret the magnitude of changes in symptom severity and substance use risk over time. Unstandardized estimates were prioritized because they preserve the original clinical and behavioral meaning of the outcome scales, allowing direct interpretation of changes in symptom severity and substance use risk. Given the pilot nature of the study, modest sample size and two-community clustered design, standardized effect size estimates were also likely to be unstable and highly sample-dependent. Therefore, the analytic emphasis was placed on effect directions, confidence intervals and longitudinal patterns of change rather than standardized magnitude estimates alone.

We conducted post-hoc pairwise comparisons of estimated marginal means to evaluate (a) within-group changes across waves and (b) between-group differences at the follow-up point. Model diagnostics, including assessments of normality of residuals, homoscedasticity and influential observations, were performed to evaluate model assumptions. As a robustness check, we compared pooled multiple imputation (MI) results with complete-case analyses; findings were substantively consistent, supporting the stability of the primary analytic approach. All analyses were conducted in Stata 19.5 (StataCorp, College Station, TX).

## Results

### Sample characteristics

At baseline, participants in the treatment group reported significantly higher levels of depressive and stress symptoms than those in the control group (*p* = 0.031 and *p* = 0.006, respectively; Table 1). This imbalance is an expected limitation of the single-cluster-per-arm design and should be considered when interpreting treatment-control differences on these outcomes; all models adjusted for baseline demographic covariates. Demographic characteristics were well balanced between groups. Overall, the mean age was 20.31 years; 54% identified as female, 81% had a secondary education or less, 70% lived with a parent or guardian and 64% were not currently employed.

**Table 1.** Baseline descriptive statistics overall and by treatment and control group Table 1. long description.

	Total^a^ (*N* = 94)	Treatment^a^ (*n* = 49)	Control^a^ (*n* = 45)	*p*-value
Outcome scores^b^				
Depression	7.05	8.15	5.85	0.031
Anxiety	6.21	6.83	5.54	0.193
Stress	19.11	21.11	16.94	0.006
Tobacco	3.18	3.27	3.07	0.870
Alcohol	13.48	13.16	13.82	0.748
Cannabis	12.62	13.30	11.87	0.539
Age	20.31	20.55	20.04	0.361
Sex				0.159
Male	0.46	0.39	0.53	
Female	0.54	0.61	0.47	
Education level				0.841
≤Secondary	0.81	0.82	0.80	
>Secondary	0.19	0.18	0.20	
Living situation				0.107
With parent/guardian	0.70	0.78	0.62	
Alone or with spouse	0.30	0.22	0.38	
Employment status				0.460
Not working	0.64	0.67	0.60	
Working	0.36	0.33	0.40	

a Means were calculated for continuous variables and proportions for binary variables.

b Outcome scales were 0–27 (depression), 0–21 (anxiety), 0–40 (stress), 0–25 (tobacco), and 0–33 for alcohol and cannabis. Higher scores indicate greater risk or symptom severity.

Of the 94 participants at baseline, 9 (10%) were lost at the 3-month assessment and 19 (20%) at the 6-month assessment. All the attrition occurred in the treatment group. Across study waves, outcome-specific missing data not attributable to attrition ranged from 0% (tobacco, alcohol and cannabis at the 6-month assessment) to 21% (depression and anxiety at the 3-month assessment). Missing data were concentrated at the 3-month assessment among participants in the treatment group, ranging from 5% for alcohol risk to 37% for depression and anxiety measures. In contrast, missingness at the 3-month assessment in the control group ranged from 0% to 11%.

### Preliminary effects of YEDI intervention on alcohol, tobacco and cannabis risk and mental health outcomes

The greatest decreases between treatment and control groups from baseline to Wave 2 were observed for depressive symptoms (*B* = −2.86, 95% CI [−5.47, −0.25]), alcohol risk (*B* = −4.94, 95% CI [−9.67, −0.20]) and cannabis risk (*B* = −5.37, 95% CI [−10.42, −0.31]) (Table 2). Smaller decreases were observed for anxiety symptoms (*B* = −1.80, 95% CI [−4.08, 0.48]) and tobacco risk (*B* = −1.45, 95% CI [−5.28, 2.37]). In contrast, stress symptoms increased from baseline to Wave 2 between treatment and control groups (*B* = 4.60, 95% CI [0.23, 8.96]).

**Table 2.** Linear mixed effects results by outcome Table 2. long description.

	Depression B (95% CI)	Anxiety B (95% CI)	Stress B (95% CI)	Tobacco B (95% CI)	Alcohol B (95% CI)	Cannabis B (95% CI)
Group (Treatment)	2.37 (0.36, 4.37)*	1.48 (−0.41, 3.38)	4.31 (1.53, 7.09)**	−0.14 (−2.76, 2.48)	−1.03 (−4.86, 2.80)	0.88 (−3.44, 5.19)
Wave						
2	0.55 (−1.49, 2.59)	−0.76 (−2.44, 0.93)	−5.92 (−9.45, −2.39)**	2.80 (0.17, 5.43)*	3.20 (−0.53, 6.92)	2.86 (−0.66, 6.38)
3	−0.80 (−2.82, 1.23)	−2.20 (−3.94, −0.45)*	−6.73 (−10.10, −3.37)***	−1.10 (−3.44, 1.23)	1.73 (−2.80, 6.25)	0.66 (−4.62, 5.93)
Wave × Group						
Wave 2 × Group	−2.86 (−5.47, −0.25)*	−1.80 (−4.08, 0.48)	4.60 (0.23, 8.96)*	−1.45 (−5.28, 2.37)	−4.94 (−9.67, −0.20)*	−5.37 (−10.42, −0.31)*
Wave 3 × Group	−1.13 (−3.63, 1.38)	0.82 (−1.42, 3.07)	5.42 (1.33, 9.50)**	2.87 (−0.61, 6.35)	−2.97 (−8.65, 2.70)	−1.52 (−8.35, 5.32)
Age	0.15 (−0.21, 0.52)	0.01 (−0.26, 0.29)	0.33 (−0.14, 0.79)	0.46 (−0.02, 0.94)	1.17 (0.60, 1.74)***	0.46 (−0.24, 1.16)
Sex (Male)	−1.61 (−2.96, −0.27)*	−1.64 (−2.71, −0.57)**	−2.04 (−4.01, −0.07)*	−0.46 (−2.51, 1.59)	−2.17 (−4.73, 0.38)	3.53 (0.57, 6.50)*
Education (>Secondary)	−1.07 (−2.52, 0.38)	−0.89 (−2.02, 0.24)	2.37 (0.31, 4.42)*	−3.53 (−5.71, −1.34)**	−0.86 (−4.21, 2.49)	−1.59 (−5.91, 2.73)
Living situation (Alone/Spouse)	−0.44 (−1.72, 0.85)	0.05 (−0.96, 1.05)	−0.31 (−2.40, 1.79)	−0.44 (−2.26, 1.38)	−0.66 (−3.51, 2.20)	1.70 (−1.41, 4.82)
Employment (Working)	0.25 (−1.01, 1.50)	−0.34 (−1.42, 0.73)	0.24 (−2.04, 2.52)	−0.51 (−2.32, 1.30)	0.45 (−2.62, 3.53)	−0.28 (−3.71, 3.15)

*p < 0.05, **p < 0.01, ***p < 0.001.

*Note:* Group (Treatment) reflects baseline differences in outcomes between treatment and control groups. Wave 2 = 3-months follow-up and wave 3 = 6-month follow-up.

Compared to the baseline-to-Wave 2 treatment differences, the baseline-to-Wave-3 treatment-control differences were smaller for depressive (*B* = −1.13, 95% CI [−3.63, 1.38]) and anxiety symptoms (*B* = 0.82, 95% CI [−1.42, 3.07]) and alcohol (*B* = −2.97, 95% CI [−8.65, 2.70]) and cannabis risk (*B* = −1.52, 95% CI [−8.35, 5.32]). There were greater increases, however, in stress symptoms (*B* = 5.42, 95% CI [1.33, 9.50]) and tobacco risk (*B* = 2.87, 95% CI [−0.61, 6.35]).

Compared with females, males reported lower levels of mental health symptoms (depression (*B* = −1.61, 95% CI [−2.96, −0.27]), anxiety (*B* = −1.64, 95% CI [−2.71, −0.57]), and stress (*B* = −2.04, 95% CI [−4.01, −0.07], *p* = 0.04)) and tobacco (*B* = −0.46, 95% CI [−2.51, 1.59]) and alcohol risk (*B* = −2.17, 95% CI [−4.73, 0.38]), but higher cannabis risk (*B* = 3.53, 95% CI [0.57, 6.50]). Participants with more than secondary education reported higher stress levels (*B* = 2.37, 95% CI [0.31, 4.42], *p* = 0.02) but lower levels of other mental health symptoms and substance use risks compared to those with less education. Differences were relatively small between individuals living alone or with a spouse and those living with parents or guardians, and between those who worked and those who did not.

Post-hoc within-group analyses revealed that differences in depressive symptoms were greatest for the control group between Waves 2 and 3 (Δ = −1.34, 95% CI [−3.25, 0.57]) and for the treatment group between baseline and Wave 2 (Δ = −2.31, 95% CI [−3.93, −0.69]) (Table 3). Relatively similar reductions were observed in the treatment group between baseline and Wave 3 (Δ = −1.92, 95% CI [−3.55, −0.30]). For anxiety symptoms, within-group differences were greatest in the control group from baseline to Wave 3 (Δ = −2.20, 95% CI [−3.94, −0.46]) and in the treatment group between baseline and Wave 2 (Δ = −2.55; 95% CI: −4.06, −1.05). The highest within-group reductions in stress symptoms were from baseline to Wave 3 (Δ = −6.73, 95% CI [−10.07, −3.35]) in the control group, with similar reductions between baseline and Wave 2 (Δ = −5.92, 95% CI [−9.45, −2.39]), and from baseline to Wave 2 in the treatment group (Δ = −1.33, 95% CI [−3.86, 1.20]).

**Table 3.** Pairwise comparisons of estimated marginal means by outcome Table 3. long description.

Comparison	Depression Δ (95% CI)	Anxiety Δ (95% CI)	Stress Δ (95% CI)	Tobacco Δ (95% CI)	Alcohol Δ (95% CI)	Cannabis Δ (95% CI)
Control: Wave 2-Baseline	0.55 (−1.49, 2.59)	−0.76 (−2.44, 0.93)	−5.92 (−9.45, −2.39)**	2.80 (0.17, 5.43)*	3.20 (−0.53, 6.92)	2.86 (−0.66, 6.38)
Control: Wave 3-Baseline	−0.80 (−2.82, 1.23)	−2.20 (−3.94, −0.45)*	−6.73 (−10.10, −3.37)***	−1.10 (−3.44, 1.23)	1.73 (−2.80, 6.25)	0.66 (−4.62, 5.93)
Control: Wave 3-Wave 2	−1.34 (−3.25, 0.57)	−1.44 (−2.85, −0.03)*	−0.81 (−3.83, 2.21)	−3.90 (−6.22, −1.58)**	−1.47 (−4.85, 1.91)	−2.20 (−6.75, 2.34)
Treatment: Wave 2-Baseline	−2.31 (−3.93, −0.69)**	−2.56 (−4.09, −1.03)**	−1.33 (−3.86, 1.20)	1.35 (−1.40, 4.10)	−1.74 (−4.65, 1.17)	−2.51 (−6.11, 1.10)
Treatment: Wave 3-Baseline	−1.92 (−3.55, −0.30)*	−1.37 (−2.89, 0.14)	−1.31 (−3.79, 1.17)	1.77 (−0.80, 4.34)	−1.25 (−4.78, 2.28)	−0.86 (−5.38, 3.66)
Treatment: Wave 3-Wave 2	0.39 (−1.10, 1.87)	1.19 (−0.14, 2.52)	0.01 (−2.67, 2.70)	0.42 (−2.89, 3.74)	0.49 (−3.21, 4.19)	1.65 (−2.69, 5.98)
Treatment-Control at Baseline	2.37 (0.36, 4.37)**	1.48 (−0.41, 3.38)	4.31 (1.53, 7.09)**	−0.14 (−2.76, 2.48)	−1.03 (−4.86, 2.80)	0.88 (−3.44, 5.19)
Treatment-Control at Wave 2	−0.49 (−2.30, 1.31)	−0.32 (−1.91, 1.27)	8.91 (5.74, 12.07)***	−1.59 (−4.92, 1.74)	−5.97 (−9.70, −2.24)**	−4.49 (−8.63, −0.35)*
Treatment-Control at Wave 3	1.24 (−0.89, 3.37)	2.31 (0.84, 3.77)**	9.73 (6.39, 13.07)***	2.73 (−0.26, 5.73)	−4.01 (−8.33, 0.32)	−0.64 (−6.37, 5.09)

*p < 0.05, **p < 0.01, ***p < 0.001.

Tobacco risk changed the most between Waves 2 and 3 (Δ = −3.90, 95% CI [−6.22, −1.58]) in the control group and between baseline and Wave 3 in the treatment group (Δ = 1.77, 95% CI [−0.80, 4.34]). Within-group differences in both alcohol and cannabis risk were greatest between baseline and Wave 2 for both the control (alcohol: Δ = 3.20, 95% CI [−0.53, 6.92]; cannabis: Δ = 2.86, 95% CI [−0.66, 6.38]) and treatment groups (alcohol: Δ = −1.74, 95% CI [−4.65, 1.17]; cannabis: Δ = −2.51, 95% CI [−6.11, 1.10]).

## Discussion

This pilot study provides preliminary evidence regarding the feasibility and potential short-term impacts of a youth-focused intervention targeting alcohol, tobacco and cannabis risk and symptoms of depression, anxiety and stress among adolescents and young adults living in urban informal settlements in Nairobi. The clearest signal in the data is also the most sobering: the intervention produced short-term reductions in depression, alcohol and cannabis risk by 3 months, but these gains did not persist to 6 months. Stress differences were driven by sharp reductions in the control group, not worsening in the treatment group, a pattern that complicates interpretation but does not undermine the short-term substance use findings. Taken together, the results suggest that brief, skills-based behavioral interventions can produce early change among youth in high-adversity informal settlement contexts, but that the structural conditions sustaining substance use and distress quickly reassert themselves once the intervention ends.

The digital component of YEDI also warrants further consideration. Although this study was not designed to isolate or test the independent effects of the mobile platform, the integration of mobile-based reminders, psychoeducation content and brief behavioral reinforcement reflected an effort to extend coping support beyond the in-person sessions. In many African settings, including Kenya, smartphones are ubiquitous, and data access is readily available (Kemp, 2016). In informal settlement settings where access to behavioral health services is limited, digitally supported models may offer a potentially scalable strategy for reinforcing intervention content, maintaining engagement and supporting continuity of care between sessions (Naslund et al., 2017; Boumparis et al., 2019; Naslund et al., 2019). At the same time, the absence of sustained effects at 6 months suggests that digital reinforcement alone may be insufficient to counteract the broader structural stressors affecting youth in these environments. Future studies should directly evaluate patterns of digital engagement, acceptability, usability and the added value of mobile reinforcement components, using designs that isolate the effects of digital and in-person interventions.

Depressive symptoms declined significantly in the treatment group across both follow-up waves. By 6 months, improvements in the treatment group were offset by parallel decreases in the control group, a convergence that makes it difficult to attribute sustained benefits to the intervention itself. This pattern does not necessarily indicate a null effect. It may instead reflect the responsiveness of depressive symptoms to non-specific social contact and structured activities, both of which the control condition partially received through the NACADA educational materials and group data-collection events. Research consistently shows that structural factors, such as poverty, unemployment, overcrowding and weak health systems, heighten exposure to psychosocial stressors, leading to adverse mental and behavioral health outcomes (Odongo and Donghui, 2021; Mukolwe et al., 2024; Okumu et al., 2025). Previous research in these communities has shown that exposure to three or more ACEs and ongoing adversities, such as violence, unemployment and unstable housing, increases depressive symptoms (Byansi et al., 2025). Thus, the short-term improvement indicates youth responsiveness to structured, supportive programs. However, the brevity of this 7-week intervention, completed prior to the 3-month assessment, likely contributed to the lack of sustained effects, suggesting that structural conditions demand more intensive or prolonged intervention models to achieve lasting improvements.

For anxiety symptoms, while differences between the treatment and control groups were small, both groups showed significant within-group decreases at different time points. The treatment group improved from baseline to Wave 2, while the control group improved from baseline to Wave 3. These findings reflect the instability of anxiety symptoms in contexts marked by daily uncertainty, community violence and lack of social and economic opportunities. Research on urban informal settlements shows that anxiety often spikes in response to environmental stressors and may temporarily decline when community-level stressors shift (Friedberg et al., 2023; Winter et al., 2023). Thus, the lack of a consistent intervention effect may reflect the overwhelming influence of contextual factors on anxiety trajectories, potentially overshadowing the intervention’s impact or requiring a more intensive dose to counteract these daily fluctuations.

Stress outcomes revealed a large Wave × Group interaction coefficient, but these effects were counterintuitive. Relative differences were driven by sharp reductions in stress within the control group, not increases in the treatment group. The treatment group’s stress levels remained relatively stable, while the control group declined substantially at 3- and 6-month follow-ups. This pattern suggests potential influences, such as community-level events or possible contamination in the control setting, that may have contributed to a reduction in reported stress. These possibilities cannot be disentangled with the current data, and this ambiguity is precisely why the stress finding should not be interpreted in isolation from the two-cluster limitation. It may also reflect differences in exposure to acute stressors between sites, consistent with research showing substantial heterogeneity across Nairobi’s informal settlements (Kibichii and Mwaeke, 2024). Critically, the treatment group’s stress did not worsen, and the intervention was not harmful. But the stress finding should not be read as evidence of a treatment effect, and it underscores why relative effects in pilot trials with few clusters require careful contextual interpretation.

Substance use outcomes showed short-term benefits from the intervention. Alcohol and cannabis risk declined significantly in the treatment group relative to the control group between baseline and 3-month follow-up. These findings align with prior research demonstrating that brief psychoeducation and behavior-change strategies can reduce early-stage substance use among youth (National Authority for the Campaign Against Alcohol and Drug Abuse (NACADA), 2022). However, these initial gains in alcohol and cannabis risk were not sustained at 6-month follow-up, suggesting that brief interventions may be insufficient in environments where these substances are widely available, inexpensive and normalized (Mmereki et al., 2022; Musyoka et al., 2025). Furthermore, tobacco risk was comparable between groups, even though the control group showed a temporary increase, a pattern that may reflect the greater complexity of tobacco use habits and stronger dependence compared to alcohol or cannabis (Mwenda et al., 2023). That tobacco use was unaffected while alcohol and cannabis showed early change may reflect meaningful differences in the nature and context of these substances: tobacco use in this population tends to be more habitual and socially embedded, and the YEDI curriculum did not include tobacco-specific content beyond general psychoeducation. The differential substance-use pattern therefore carries implications for intervention content rather than simply for duration or intensity.

## Limitations

Several limitations must be acknowledged. First, attrition occurred entirely within the treatment group and missingness was highest at 6-month follow-up, reducing power to detect sustained effects. The concentration of attrition in the treatment group also provides important insights into implementation and retention for intervention research in informal settlements. High residential mobility, unstable employment, competing economic demands and inconsistent phone access may have limited sustained engagement over time. Because dropout may be associated with non-response to treatment, the MAR assumption underlying our multiple imputation approach may not fully hold; findings at 6 months should therefore be interpreted with particular caution. Future studies should incorporate more intensive retention strategies, including transportation support, flexible scheduling, ongoing peer outreach, community-based follow-up systems and booster sessions. Second, the imbalance in missing data across groups may introduce residual bias even after multiple imputation. Third, only two communities were included with one cluster per condition. This design prevented reliable estimation of between-cluster variance and may have resulted in underestimated standard errors and an increased risk of Type I error. Consequently, some observed differences may reflect settlement-specific contextual factors rather than intervention effects. The findings should therefore be viewed as exploratory and hypothesis-generating rather than confirmatory. Future trials should include substantially more clusters to allow robust cluster-level inference. Fourth, several outcomes improved in both groups, suggesting that unmeasured community-level events or seasonal factors may have influenced trajectories. Fifth, the sample size was small, and the study was not powered to detect definitive intervention effects. Although attendance, retention, and facilitator competency provided indirect indicators of feasibility, formal measures of acceptability, appropriateness, fidelity and digital engagement were not collected. Consequently, conclusions regarding implementation processes are limited. Future studies should incorporate standardized implementation outcomes and measures of participant engagement with the mobile platform. Finally, outcomes were self-reported, raising the possibility of recall or social desirability bias (e.g., participants may have underreported alcohol use or other stigmatized behaviors).

## Conclusions

YEDI produced meaningful preliminary short-term reductions in depression, alcohol use and cannabis risk among adolescents and young adults living in Nairobi’s informal settlements. That these gains were not sustained at 6 months does not diminish their significance. This pilot study clarifies what a brief, seven-session intervention can and cannot reasonably accomplish in contexts shaped by poverty, violence exposure and severely constrained services. The study’s two-cluster design limits causal inference and future trials need more communities, longer follow-up periods and retention strategies tailored to high-mobility populations. Future models should integrate YEDI’s core skills with material supports and sustained peer contact. This pilot provides the feasibility data and effect estimates needed to design and power the next generation of trials to improve the well-being of vulnerable young people in SSA.

## Data Availability

The data presented in this study and the STATA code used for the analyses are available on request from the corresponding author.

## Long descriptions

### Figure 1. Long description

A horizontal flowchart organized into three vertical layers.

Top Layer: A thin grey header bar labeled Group C B T (G C B T) with bullet points for 7 weekly sessions, approximately 60 min/session, 8 to 10 participants, 2 trained lay facilitators, and Community settings.

Middle Layer: Four phase banners containing seven numbered session boxes connected by right-pointing arrows.

1. Engagement Phase: Session 1, Introduction to Y E D I & Building Group Cohesion. Focuses on Engagement & Alliance, group norms, rapport, and psychosocial safety.

2. Psychoeducation Phase: Session 2, Psychoeducation: Substance Use & Mental Health. Focuses on Psychoeducation regarding alcohol, cannabis, tobacco, mental health, and help-seeking.

3. Skills Building Phase (shaded grey): Includes three sessions.

- Session 3: Relaxation for Stress Management. Focuses on Affect Regulation, breathing, progressive relaxation, and grounding.

- Session 4: Problem-Solving Skills. Focuses on Behavioural Skills, structured problem-solving, stressor I D, and solutions.

- Session 5: Cognitive Restructuring. Focuses on Cognitive Skills, maladaptive thoughts, and belief-emotion-behaviour relationship.

4. Consolidation Phase: Includes two sessions.

- Session 6: Relapse Prevention & Life Skills. Focuses on Maintenance & Generalisation, triggers, coping plans, communication, and social support.

- Session 7: Feedback, Celebrations & Closing Ceremony. Focuses on Consolidation & Closure, skill review, progress celebration, and goal-setting.

Bottom Layer: A dashed-line box spanning the width of the chart labeled Mobile Platform Reinforcement (yedi dot co dot ke). It lists between-session reminders, psychoeducational content, and animated skill demonstrations, noting it is active throughout all 7 weeks.

Navigate back to Figure 1..

### Table 1. Long description

The table compares a Total group (N = 94), a Treatment group (n = 49), and a Control group (n = 45).

Outcome scores (means):

* Depression: Total 7.05, Treatment 8.15, Control 5.85, p-value 0.031.

* Anxiety: Total 6.21, Treatment 6.83, Control 5.54, p-value 0.193.

* Stress: Total 19.11, Treatment 21.11, Control 16.94, p-value 0.006.

* Tobacco: Total 3.18, Treatment 3.27, Control 3.07, p-value 0.870.

* Alcohol: Total 13.48, Treatment 13.16, Control 13.82, p-value 0.748.

* Cannabis: Total 12.62, Treatment 13.30, Control 11.87, p-value 0.539.

Demographics and Characteristics:

* Age (mean): Total 20.31, Treatment 20.55, Control 20.04, p-value 0.361.

* Sex (proportions, p-value 0.159): Male (Total 0.46, Treatment 0.39, Control 0.53), Female (Total 0.54, Treatment 0.61, Control 0.47).

* Education level (proportions, p-value 0.841): Less than or equal to Secondary (Total 0.81, Treatment 0.82, Control 0.80), Greater than Secondary (Total 0.19, Treatment 0.18, Control 0.20).

* Living situation (proportions, p-value 0.107): With parent or guardian (Total 0.70, Treatment 0.78, Control 0.62), Alone or with spouse (Total 0.30, Treatment 0.22, Control 0.38).

* Employment status (proportions, p-value 0.460): Not working (Total 0.64, Treatment 0.67, Control 0.60), Working (Total 0.36, Treatment 0.33, Control 0.40).

Navigate back to Table 1..

### Table 2. Long description

The table presents B coefficients and 95 percent C I for six outcomes.

Column headers: Depression, Anxiety, Stress, Tobacco, Alcohol, and Cannabis.

Rows and key data points:

* Group (Treatment): Significant positive effects for Depression (2.37) and Stress (4.31).

* Wave 2: Significant decrease in Stress (-5.92) and increase in Tobacco (2.80).

* Wave 3: Significant decrease in Anxiety (-2.20) and Stress (-6.73).

* Wave 2 x Group: Significant negative interaction for Depression (-2.86), Alcohol (-4.94), and Cannabis (-5.37); significant positive interaction for Stress (4.60).

* Wave 3 x Group: Significant positive interaction for Stress (5.42).

* Age: Significant positive effect for Alcohol (1.17).

* Sex (Male): Significant negative effects for Depression (-1.61), Anxiety (-1.64), and Stress (-2.04); significant positive effect for Cannabis (3.53).

* Education (>Secondary): Significant positive effect for Stress (2.37) and significant negative effect for Tobacco (-3.53).

* Living situation and Employment: No significant effects across any outcomes.

Statistical significance is denoted by asterisks: * p < 0.05, ** p < 0.01, *** p < 0.001.

Navigate back to Table 2..

### Table 3. Long description

The table consists of 7 columns and 9 rows of data. The columns are Comparison, Depression, Anxiety, Stress, Tobacco, Alcohol, and Cannabis. Each cell contains a Delta value followed by a 95 percent C I in brackets.

Row 1: Control Wave 2 minus Baseline. Stress shows a significant decrease of minus 5.92. Tobacco shows a significant increase of 2.80.

Row 2: Control Wave 3 minus Baseline. Anxiety shows a significant decrease of minus 2.20. Stress shows a significant decrease of minus 6.73.

Row 3: Control Wave 3 minus Wave 2. Anxiety shows a significant decrease of minus 1.44. Tobacco shows a significant decrease of minus 3.90.

Row 4: Treatment Wave 2 minus Baseline. Depression shows a significant decrease of minus 2.31. Anxiety shows a significant decrease of minus 2.56.

Row 5: Treatment Wave 3 minus Baseline. Depression shows a significant decrease of minus 1.92.

Row 6: Treatment Wave 3 minus Wave 2. No significant changes reported.

Row 7: Treatment minus Control at Baseline. Significant differences in Depression 2.37 and Stress 4.31.

Row 8: Treatment minus Control at Wave 2. Significant differences in Stress 8.91, Alcohol minus 5.97, and Cannabis minus 4.49.

Row 9: Treatment minus Control at Wave 3. Significant differences in Anxiety 2.31 and Stress 9.73.

Significance levels are indicated by asterisks: one asterisk for p less than 0.05, two for p less than 0.01, and three for p less than 0.001.

Navigate back to Table 3..
