# Supplementary material for: Preliminary effectiveness of the youth empowerment digital intervention (YEDI): A pilot clustered community study of a group cognitive behavioral therapy intervention with digital reinforcement among youth in Kenya’s informal settlements
Source: Glob Ment Health (Camb). 2026 Jul 21;13:e170. doi: 10.1017/gmh.2026.10278 (PMC13458862; doi:10.1017/gmh.2026.10278)
Supplement: Peer review history

# Reviewer report: Review: Preliminary effectiveness of the youth empowerment digital intervention (YEDI): A pilot clustered community study of a group cognitive behavioral therapy intervention with digital reinforcement among youth in Kenya’s informal settlements — R0/PR2

**DOI:** 10.1017/gmh.2026.10278.pr2

**Authors:** Munyaradzi Madhombiro

**Affiliations:** University of Zimbabwe, Zimbabwe

Review GMH-2026-0103

Abstract

The abstract is written well

Introduction

Although the introduction reads well, please attend to, Line 34, please correct the spelling ‘crucil’ for ‘crucial’.

Materials and Methods

1.It is difficult to understand how two settlements will be randomly assigned to intervention and control conditions especially in a cluster randomized trial. One would aim at recruiting from several clusters and at a minimum, 10-15 clusters is required to ensure statistical validity.

2.The sampling here, snowball creates multiple biases including selection, network centrality and homogeneity, how was this dealt with? Additionally, the fact that this was a cluster trial, with just two clusters, how did the researchers mitigate against this?

3.In study conditions, please maintain the same font, for control and intervention!

4.Line 33-34 suggest that the control arm also received elements of the YEDI intervention on an app? If so this means the control arm was also active, what are the implications of this? Would this boost the outcomes in this arm? Please explain the rationale for this approach?

5.The intervention content and delivery are described well and adequately.

6.Given that at the outset, the study was not assessing the effectiveness of the intervention, but the feasibility, the reporting should therefore emphasize that.

Results

It is clear that the study had significant loss to followup, which speaks to the feasibility of the study as the main output of the study, so what are the implications of this? And future studies, how can they mitigate against this in similar settings?

Discussion

Again, given that the study main purpose was explore the feasibility, and not powered adequately, is there enough data findings to speak to the effectiveness of YEDI? The authors should keep their focus on the feasibility of the intervention, for which the study design can answer!

Limitations

1.Accepted that the study is not powered enough, the design is also not appropriate, especially given that it was a cluster, but with only two clusters. This is a major limitation of the study. Also worrying is the sampling frame, especially that it was snowballing, and the associated biases mentioned above, it is even more concerning.

# Reviewer report: Review: Preliminary effectiveness of the youth empowerment digital intervention (YEDI): A pilot clustered community study of a group cognitive behavioral therapy intervention with digital reinforcement among youth in Kenya’s informal settlements — R0/PR3

**DOI:** 10.1017/gmh.2026.10278.pr3

The manuscript is good and i recommend it to be published

# Reviewer report: Review: Preliminary effectiveness of the youth empowerment digital intervention (YEDI): A pilot clustered community study of a group cognitive behavioral therapy intervention with digital reinforcement among youth in Kenya’s informal settlements — R0/PR4

**DOI:** 10.1017/gmh.2026.10278.pr4

This article reports results of a clustered randomized controlled trial of a substance use and depression intervention for youth in Kenya. Results are very important to the literature, as the researchers implemented an evidence-based approach, including digital supports, and found mixed results without sustained effects on primary outcomes. They insightfully make the point that addressing structural factors is likely needed in addition to evidence-based mental health components. Publishing these types of mixed results is important for pushing the field forward in understanding the potential and limitations of the common approaches used in global mental health.

There are several areas that would benefit from clarification and checks for consistency.

Abstract:

- It would be helpful to clarify whether this is a prevention or treatment intervention and whether the sample had depression and substance use symptoms; the criteria only mention being under a certain ASSIST score.

- The abstract should be clearer that this study was not fully powered for a test of efficacy and that all effects and null results should be interpreted as preliminary.

Impact Statement: Authors should consider adding the point that these results push the field to consider the likely need for structural interventions. Conversely, it wasn’t clear that results underscore the importance of the digital element, as the design of this study did not test the addition of that element, and results were not sustained despite the digital approach.

Introduction:

- Authors provide helpful context and highlight the gaps in substance use and mental health treatment for youth. However, it would be helpful to review the programs that have been tested in SSA (even if they do have limitations, such as not being adapted or not being tested yet with randomized designs) and summarize their results.

- The intervention description is not clear. Authors describe group CBT in a way that doesn’t describe all forms of CBT delivered in groups and there are not citations to help understand if it is referring to a specific model that they used as a foundation for YEDI. Authors then label the specific intervention in this study as YEDI without describing it clearly.

- Major: The acronym YEDI stands for different words in different places (Youth Empowerment and Development Initiative vs. the title, Youth Empowerment Digital Intervention. Authors should clarify, including whether the digital element is a core focus. It would also be helpful to know how it was developed and/or adapted given the limitations the team highlights for previous interventions. The digital aspect also is not mentioned. In addition to adding description within this manuscript, it would be helpful to cite any other resources or publications where the intervention is described in more detail.

Methods:

- Snowball sampling seems appropriate, though it would be helpful to know more detail about how the target sample was described to the CHPs and the youth—and more about how the CHPs identified the original three youth. For example, was depression mentioned at all as something they should consider in identifying youth or referring their peers, or just substance use? How was mild/moderate described to them to try to recruit those with the appropriate level of need? (These details are helpful for contextualizing results but also helpful for future research that may benefit from this method.)

- Related, later, Methods state that “Recruitment was facilitated through partnerships with two youth-serving community-based organizations. RAs and youth mobilizers distributed study information and screened interested participants for eligibility.” How did this fit with the CHP method described above?

- After seeing the differences between substance use and depression between the two clusters at baseline, I was curious to learn more about how the communities were selected and to see descriptions of each of them. The rationale for cluster randomization would also be valuable to explain since the intervention is not delivered at a community level. (It also seemed unexpected that all attrition for assessments occurred in the treatment group and that the stress in the control group decreased so much. Both raise questions about community-level differences.)

- Was there a minimum ASSIST score? It is unclear whether this was recruited to be a substance using population. Authors should also explicitly state and explain why depression was a main outcome (and is often the main target of CBT) but seemingly not considered during recruitment.

- More is needed on who the selected facilitators were, including ages and levels of background and experience. The use of ENACT is a strength; citations for EnACT should be included.

- The intervention description is slightly better in Methods, but there is still no description of the development process and only one sentence on the digital component.

- Measures:

o Minor: For education level, it says categories were less than secondary vs. higher than secondary; clarify if stopping after secondary went in the second category.

o In the power analysis section, this is described as focused on feasibility/acceptability, though there are no measures of feasibility or acceptability described in the Measures section and Results/Discussion focus on effectiveness data. Throughout, it seems more accurate to say that this was a pilot effectiveness trial to understand preliminary effects, rather than mentioning feasibility and acceptability as the focus.

o Statistical analysis: Methods state analyses were focused on estimating effect sizes and examining patterns of change, but methods and results seem focused on significance testing without including standardized effect sizes.

Results:

- Authors should consider adding “preliminary” to the subheading that now says “effects of…”

- Authors should consider reporting standardized effect sizes or explain why that approach wasn’t chosen.

- Related to methods comment above, the results still seem to emphasize significance testing rather than the effect sizes and patterns of change that are emphasized in the power analysis section.

Discussion: The discussion is helpful in summarizing results and expands the discussion of the more subtle patterns, including concerns about the site-level differences that could have influenced results. More on standardized effect sizes would be useful, depending on any related revisions to the analysis or results. There is no discussion related to feasibility, acceptability, or observations related to the digital aspect, which could be expanded.

# Editor report: Recommendation: Preliminary effectiveness of the youth empowerment digital intervention (YEDI): A pilot clustered community study of a group cognitive behavioral therapy intervention with digital reinforcement among youth in Kenya’s informal settlements — R0/PR5

**DOI:** 10.1017/gmh.2026.10278.pr5

**Affiliations:** Psychology, University of Washington, United States

Thank you for submitting your work for consideration to Cambridge Prisms: Global Mental Health. I have secured reviews from 3 external peer reviewers. I have also reviewed your manuscript myself. Myself and the reviewers agree that this is important work and has the potential to make an impact on the field. However, some edits may help to strengthen its contribution. I would particularly call attention to comments from Reviewers 1 and 3 about ensuring the results and discussion emphasize the pilot and feasibility nature of you work as to not unintentionally overstate findings.

# Editor report: Decision: Preliminary effectiveness of the youth empowerment digital intervention (YEDI): A pilot clustered community study of a group cognitive behavioral therapy intervention with digital reinforcement among youth in Kenya’s informal settlements — R0/PR6

**DOI:** 10.1017/gmh.2026.10278.pr6

**Affiliations:** London School of Hygiene & Tropical Medicine, United Kingdom of Great Britain and Northern Ireland

No accompanying comment.

# Reviewer report: Review: Preliminary effectiveness of the youth empowerment digital intervention (YEDI): A pilot clustered community study of a group cognitive behavioral therapy intervention with digital reinforcement among youth in Kenya’s informal settlements — R1/PR8

**DOI:** 10.1017/gmh.2026.10278.pr8

Authors have substantially improved the manuscript. I have three remaining comments for authors and editors to consider:

1. The abstract results and conclusions still read like an effectiveness trial with focus on the clinical outcomes rather than feasibility, proof-of-concept, and very preliminary indicators of clinical effectiveness. Given limitations of the design, it cannot be concluded the YEDI produced these outcomes, though it can certainly be stated that short-term results are promising.

2. Minor/Optional: The title of the paper and name of the intervention imply a strong focus on the digital component. The intervention description is now much clearer in the paper. This is just an observation for authors to consider, as readers who quickly see the title in searches without reading further might assume this is primarily a digital approach.

3. Authors may consider more formally reporting any feasibility or acceptability data given that this is best designed as a proof-of-concept. Attendance/attrition is one indicator, but if there are other indicators, including EnACT competency results, that would further strengthen the manuscript. If no formal measures of these are available, authors should note this as a limitation that future research could address by including more implementation process assessment.

# Reviewer report: Review: Preliminary effectiveness of the youth empowerment digital intervention (YEDI): A pilot clustered community study of a group cognitive behavioral therapy intervention with digital reinforcement among youth in Kenya’s informal settlements — R1/PR9

**DOI:** 10.1017/gmh.2026.10278.pr9

**Authors:** Munyaradzi Madhombiro

**Affiliations:** University of Zimbabwe, Zimbabwe

Given the sampling framework and the study design, the authors need to attend to the limitations. Given that there were only two clusters, the study would have increased risk of Type I errors which require mentioning.

# Editor report: Recommendation: Preliminary effectiveness of the youth empowerment digital intervention (YEDI): A pilot clustered community study of a group cognitive behavioral therapy intervention with digital reinforcement among youth in Kenya’s informal settlements — R1/PR10

**DOI:** 10.1017/gmh.2026.10278.pr10

**Affiliations:** Psychology, University of Washington, United States

I agree with the reviewers that your edits have substantially improved the manuscript. There may also be a few minor places to improve your manuscript.

# Editor report: Decision: Preliminary effectiveness of the youth empowerment digital intervention (YEDI): A pilot clustered community study of a group cognitive behavioral therapy intervention with digital reinforcement among youth in Kenya’s informal settlements — R1/PR11

**DOI:** 10.1017/gmh.2026.10278.pr11

**Affiliations:** London School of Hygiene & Tropical Medicine, United Kingdom of Great Britain and Northern Ireland

No accompanying comment.

# Editor report: Recommendation: Preliminary effectiveness of the youth empowerment digital intervention (YEDI): A pilot clustered community study of a group cognitive behavioral therapy intervention with digital reinforcement among youth in Kenya’s informal settlements — R2/PR13

**DOI:** 10.1017/gmh.2026.10278.pr13

**Affiliations:** Psychology, University of Washington, United States

No accompanying comment.

# Editor report: Decision: Preliminary effectiveness of the youth empowerment digital intervention (YEDI): A pilot clustered community study of a group cognitive behavioral therapy intervention with digital reinforcement among youth in Kenya’s informal settlements — R2/PR14

**DOI:** 10.1017/gmh.2026.10278.pr14

**Affiliations:** London School of Hygiene & Tropical Medicine, United Kingdom of Great Britain and Northern Ireland

No accompanying comment.
